# Risk stratification system to predict recurrence of intrahepatic cholangiocarcinoma after hepatic resection

**DOI:** 10.1186/s12885-017-3464-5

**Published:** 2017-07-03

**Authors:** Seogsong Jeong, Qingbao Cheng, Lifeng Huang, Jian Wang, Meng Sha, Ying Tong, Lei Xia, Longzhi Han, Zhifeng Xi, Jianjun Zhang, Xiaoni Kong, Jinyang Gu, Qiang Xia

**Affiliations:** 1 Department of Liver Surgery, Renji Hospital, School of Medicine, Shanghai Jiao Tong University, Dongfang Road, NO. 1630, Shanghai, 200127 China; 2 Department of Biliary Surgery, Eastern Hepatobiliary Surgery Hospital, Second Military Medical University, Shanghai, China; 3 Department of Biliary and Pancreatic Surgery, Shanghai East Hospital, School of Medicine, Tongji University, Shanghai, 200120 China

**Keywords:** Risk stratification, Staging system, Nomogram, Hepatitis B virus-associated intrahepatic cholangiocarcinoma, Hepatic resection

## Abstract

**Background:**

Previous nomograms for intrahepatic cholangiocarcinoma (ICC) were conducted to predict overall survival, which could be influenced by various factors. Herein, we conducted our nomogram to predict recurrence of the tumor only after hepatic resection.

**Methods:**

The nomogram was established with prognostic factors for the relapse-free survival (RFS) analyzed from our single center cohort and was evaluated by comparing with the American Joint Committee on Cancer (AJCC) staging system for the predictive accuracy.

**Results:**

Seropositivity of hepatitis B surface antigen (hazard ratio [HR], 0.505; 95% confidence interval [CI], 0.279 to 0.914; *P* = 0.024), tumor size of larger than 5 cm (HR, 1.947; 95% CI, 1.177 to 3.219; *P* = 0.009), Child-Pugh score of B (HR, 3.067; 95% CI, 1.293 to 7.275; *P* = 0.011), and lymph node metastasis (HR, 2.790; 95% CI, 1.628 to 4.781; *P* < 0.001) were found to be independent prognostic factors that significantly affected RFS. The calibration curve for the prediction revealed excellent agreement between estimation by our stratification system and actual RFS. The concordance C index of the nomogram (0.71; 95% CI, 0.65 to 0.77) revealed to be significantly higher than the AJCC staging system (0.66; 95% CI, 0.60 to 0.72). In the validation cohort, our risk stratification system (C-index 0.65; 95% CI, 0.59 to 0.71) also revealed more precise prediction than the AJCC staging system (C-index, 0.57; 95% CI, 0.50 to 0.64).

**Conclusions:**

Our nomogram could more accurately predict recurrence of ICC after hepatic resection than the AJCC staging system.

## Background

Intrahepatic cholangiocarcinoma (ICC) is the second most common primary liver cancer accounting for 15 to 20% of all primary liver malignancies [1]. It is a relatively rare disease with dismal prognosis due to frequent recurrence after surgical operation, lymph node metastasis, and rapid progression of the tumor, and 5-year mortality of patients who received surgical treatments remains higher than 50% [2]. Therefore, the interest for ICC has been rising since the incidence and mortality have been increasing [3].

To date, hepatic resection remains the most frequent option for patients with ICC to prolong their survival. However, numerous patients present with unresectable ICC at the time of diagnosis due to lack of typical clinical manifestation and rapid progression of the tumor [4]. Moreover, patients with underlying liver diseases, such as liver cirrhosis, are very common where hepatitis B virus (HBV) infection is endemic, so that a majority of patients have no chance to receive hepatic resection. To date, several evaluation procedures for resection range in cirrhotic patients have already been developed and proved to be effective. However, another important factor that needs to be focused is recurrence of the tumor which significantly reduce survival outcomes. Therefore, in order to identify who should be treated by hepatic resection, it is vital to comprehensively confirm factors that are associated with recurrence of the tumor.

Precise prediction of prognosis is important for patients as well as physicians to consider postoperative management. According to the seventh American Joint Committee on Cancer (AJCC) cancer staging manual, the staging system for ICC was separated from hepatocellular carcinoma [5]. In spite of the modification, the predictive accuracy of conventional AJCC staging system was found to be lower in Eastern population which was validated by Chinese nomogram (concordance index [C-index]: 0.65 vs. 0.74) [6]. However, the nomogram was established on the overall survival of patients, indicating that the nomogram could be influenced by a number of confounding factors rather than the recurrence of the tumor, rendering us to interpret that currently available nomograms are insufficient to accurately predict relapse of ICC after hepatic resection on the basis of the Eastern population. Therefore, we conducted our nomogram to speculate risk index to precisely predict the recurrence of ICC based on our single center experience.

## Methods

### Study patients

From Jan 2007 to Jul 2015, a total of 106 patients with ICC who underwent hepatic resection at Renji Hospital, School of Medicine, Shanghai Jiao Tong University, China were included according to the following criteria: (1) patients with pathological confirmation of mass-forming ICC from liver specimens, (2) patients with full records of clinicopathological data, (3) patients who received hepatic resection with regional lymph node dissection, and (4) patients without distant metastasis, and the included patients were enrolled in generating risk stratification system to predict recurrence of ICC, which we named “Renji nomogram”. Patients who died within 30 days upon surgical operation were excluded so as to improve the accuracy of relapse-free survival (RFS). All patients underwent radical hepatic resection (surgical margin ≥2 cm). The validation cohort of our risk stratification system included 91 patients from Eastern Hepatobiliary Surgery Hospital and 15 patients from Shanghai East Hospital who underwent hepatic resection, using the same inclusion criteria.

### Data collection

Preoperative evaluation of the patients, including a baseline history, serological examinations, and imaging studies (computed tomography of abdomen and radiography of the chest) were performed routinely. Tumor-related variables were collected by imaging studies. None of the patients received neoadjuvant therapy. Major resection was defined as hemihepatectomy or more extended resection. Regional lymph node dissection was performed in all patients. Lymph node metastasis and vascular involvement were evaluated based on preoperative imaging studies, intraoperative exploration, and postoperative pathology.

All patients were followed up in the clinic at regular intervals, and those with unavailable data in the clinic were visited by telephone inquiries. Date of last follow-up investigation and recurrence of the tumor were collected for all patients. The follow-up was performed ranging from 16 months to 118 months (median, 41 months). The primary endpoint of this study was recurrence of the tumor.

### Staging systems

Edmondson-Steiner criteria was applied in the description of histopathologic differentiation of tumors (I: well-differentiated, II: moderately differentiated, and III: poorly differentiated) [7]. The patients were scored according to the Child-Pugh scoring system, which includes total bilirubin, albumin, prothrombin time, ascites, and hepatic encephalopathy [8]. The patients were grouped according to the seventh edition of the AJCC cancer staging manual, which focused on number and size of tumors, vascular invasion, and lymph node metastasis (stage 1, T1N0M0; stage 2, T2N0M0; stage 3, T3N0M0; stage 4A, T4N0M0 or T[any]N1 M0; stage 4B, T[any]N[any]M1), to evaluate accuracy of the nomogram [5].

### Statistical analysis

Statistical analyses were carried out using SPSS, version 15.0 (Chicago, IL) and R, version 3.3.1, software packages (http://www.r-project.org/). Clinicopathological characteristics were evaluated by univariate and multivariate analyses (Cox regression). Variables were described with median (minimum to maximum) with interquartile range (IQR) or percentages. Evaluation of variables were described as hazard ratio (HR) with 95% confidence interval (CI). The power analyses were performed using “PS: Power and Sample Size Calculation” (http://biostat.mc.vanderbilt.edu/wiki/Main/PowerSampleSize) [9]. Survival curves were performed using Kaplan-Meier method. Statistically significant factors (*P* < 0.05) from the multivariate analysis were entered into the nomogram. The evaluation of the nomogram was performed through discrimination shown by the Harrell C index and a calibration plot with a bootstrapped sample.

## Results

### Clinicopathological characteristics

Baseline characteristics of the entire cohort is presented in Table 1. Approximately a half of the cases were advanced aged (*n* = 50 [47.2%]), male (*n* = 62 [58.5%]), tumor size >5 cm (*n* = 61 [57.5%]), and poorly differentiated (*n* = 54 [50.9%]) with lymph node metastasis (*n* = 58 [54.7%]). Patients received either major hepatectomy (*n* = 58 [54.7%]) or minor hepatectomy (*n* = 48 [45.3%]). A majority of patients were hepatitis B surface antigen (HBsAg)-negative (*n* = 76 [71.8%]) and not producing alpha fetoprotein (AFP) (*n* = 78 [73.6%]) with elevated serum carbohydrate antigen (CA) 19-9 (*n* = 73 [68.9%]). None of the patients were found to be seropositive for hepatitis C virus (HCV). Most patients had a single tumor (*n* = 89 [84.0%]) with Child-Pugh score of A (*n* = 100 [94.3%]). Almost a half of the patients (*n* = 54 [50.9%]) presented with poorly differentiated tumor. Vascular invasion and lymph node metastasis were found in 28 (26.4%) and 48 (45.3%) patients, respectively.

**Table 1:** Baseline characteristics of the entire cohort

Variables	Patients (%)
Age(years)[IQR]	60(35-82)[16]
Gender(female)	44(41.5)
HBsAg	30(28.2)
HBcAb(HBsAg-negative)	12(11.3)
HCV infection	0
Liver cirrhosis	25(23.6)
AFP(ng/ml)[IQR]	4.1(0.98-3000)[7.43]
CA19-9(U/ml)[IQR]	63.1(0.37-32,870)[335]
Minor hepatectomy	48(45.3)
Hemihepatictomy	47(44.3)
Expanded hepatictomy	11(10.4)
Tumor size(cm)[IQR]	6(0.8-15.0)[5.0]
Single tumor	89(84.0)
Multiple tumor	17(16.0)
Child-Pugh A	100(94.3)
Child-Pugh B	6 (5.7)
Child-Pugh C	0
Histologic differentiation	
Well or moderate	52(49.1)
Poor	54(50.9)
Vascular invasion	28(26.4)
Lymph node metastasis	48(45.3)

*IQR* interquartile range, *AFP* alpha fetoprotein, *CA19-9* carbohydrate antigen 19-9

### Independent prognostic factors

The results of the univariate and multivariate analyses are presented in Table 2. Multivariate analyses indicated that seropositivity of HBsAg (HR, 0.505; 95% CI, 0.279 to 0.914; *P* = 0.024), tumor size (HR, 1.947; 95% CI, 1.177 to 3.219; *P* = 0.009), Child-Pugh score (HR, 3.067; 95% CI, 1.293 to 7.275; *P* = 0.011), and lymph node metastasis (HR, 2.790; 95% CI, 1.628 to 4.781; *P* < 0.001) were independent prognostic factors for recurrence of ICC. However, due to limited number of cases, one variable (Child-Pugh score) revealed to be underpowered (0.442) in the present regression model.

**Table 2:** Univariate and multivariate analyses for the relapse-free survival of patients with ICC

Variables	n (%)	Relapse-free survival (%)			Univariate analysis		Multivariate analysis	
		1-year	3-year	5-year	HR (95% CI)	*P* value	HR (95% CI)	*P* value
Age								
> 60 years	50 (47.2)	36.0	15.6	11.6	1.302 (0.827-2.049)	0.253	NA	NA
≤ 60 years	56 (52.8)	42.9	25.0	9.8				
Gender								
Male	62 (58.5)	38.7	17.0	10.0	1.129 (0.711-1.793)	0.608	NA	NA
Female	44 (41.5)	40.9	25.0	11.8				
HBV infection								
HBsAg								
Present	30 (28.2)	63.3	44.0	25.0	0.424 (0.239-0.751)	0.002	0.505 (0.279-0.914)	0.024
Absent	76 (71.8)	30.3	11.8	6.3				
HBsAg-negative								
HBcAb-positive								
Present	12 (11.3)	41.7	0	0	0.734 (0.348-1.549)	0.415	NA	NA
Absent	94 (88.7)	30.0	15.0	8.9				
Liver cirrhosis								
Present	25 (23.6)	44.0	15.0	5.6	0.888 (0.517-1.526)	0.668	NA	NA
Absent	81 (76.4)	38.3	21.9	12.1				
Preoperative AFP								
> 9 ng/ml	28 (26.4)	39.3	33.3	20.8	0.889 (0.527-1.499)	0.658	NA	NA
≤ 9 ng/ml	78 (73.6)	39.7	15.2	6.7				
Preoperative CA19-9								
> 35 U/ml	73 (68.9)	34.2	14.3	9.8	1.499 (0.897-2.504)	0.119	NA	NA
≤ 35 U/ml	33 (31.1)	51.5	33.3	13.0				
Hepatectomy								
Minor	48 (45.3)	47.9	26.3	15.2	1.560 (0.975-2.494)	0.061	NA	NA
Major	58 (54.7)	32.8	16.4	7.8				
Tumor size								
> 5 cm	61 (57.5)	26.2	12.5	5.7	2.044 (1.258-3.321)	0.003	1.947 (1.177-3.219)	0.009
≤ 5 cm	45 (42.5)	57.8	32.4	19.4				
Tumor number								
Single	89 (84.0)	43.8	23.7	13.2	1.752 (1.002-3.064)	0.046	NS	NS
Multiple	17 (16.0)	17.6	5.9	0				
Child-Pugh score								
A	100 (94.3)	41.0	21.8	11.5	2.698 (1.158-6.282)	0.017	3.067 (1.293-7.275)	0.011
B	6 (5.7)	16.7	0	0				
Histology								
Well or moderate	52 (49.1)	46.2	18.2	9.8	1.084 (0.689-1.706)	0.727	NA	NA
Poor	54 (50.9)	33.3	22.4	11.6				
Vascular invasion								
Present	28 (26.4)	35.7	12.0	4.3	1.286 (0.782-2.116)	0.321	NA	NA
Absent	78 (73.6)	41.0	23.5	13.1				
Lymph node metastasis								
Present	48 (45.3)	18.8	2.2	0	2.816 (1.747-4.538)	< 0.001	2.790 (1.628-4.781)	< 0.001
Absent	58 (54.7)	56.9	37.5	22.5				

*HR* hazard ratio, *CI* confidence interval, *NA* not applicable, *HBV* hepatitis B virus, *HBsAg* hepatitis B surface antigen, *HBcAb* hepatitis B core antibody, *AFP* alpha fetoprotein, *CA19-9* carbohydrate antigen 19-9, *NS* not significant

### Recurrence of the tumor

Of the entire cohort, 1., 3., and 5-year. recurrence rates of the tumor were 60.4, 79.6, and 89.3%, respectively. Among the patients with seropositivity for HBsAg, 1., 3., and 5-year. recurrence rates were 36.7, 56.0, and 75.0%, respectively. For the patients with tumor size larger than 5 cm, 1., 3., and 5-year. recurrence occurred in 73.8, 87.5, and 94.3% of the patients, respectively. In 6 patients with Child-Pugh score of B, recurrence of the tumor was detected in 5 patients (83.3%) within the first year after surgery, and the other patient occurred recurrence at the 15th month after surgery. As for patients with lymph node metastasis, 1., 3., and 5-year. recurrence rates were 81.3, 97.8, and 100%, respectively.

### Renji nomogram

**Fig. 1:**
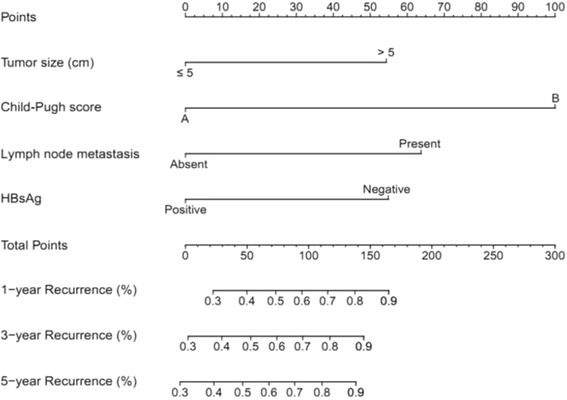
Nomogram to predict recurrence of intrahepatic cholangiocarcinoma after hepatic resection

**Fig. 2:**
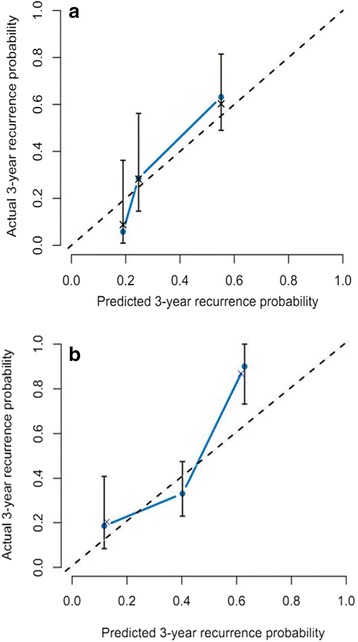
The calibration curve for predicting relapse-free survival at 3-yrs in the primary cohort (**a**) and the validation cohort (**b**)

A nomogram model to predict recurrence of ICC in patients who underwent hepatic resection is described in Fig. 1. The nomogram was established based on the 4 independent prognostic factors. The calibration plot for the accuracy of 3-year. RFS after hepatic resection revealed an ideal agreement between the prediction and actual RFS of the patients (Fig. 2a). The C-index for RFS estimation was 0.71 (standard deviation [SD], 0.06). As shown in Table 3, the nomogram was capable to stratify the patients into 4 stages (stage 1 [estimated RFS], 50 to 100%; stage 2 [estimated RFS], 30 to 50%; stage 3 [estimated RFS], 20 to 30%; stage 4 [estimated RFS], 0 to 20%).

**Table 3:** Points per unit of linear predictor for RFS of ICC

Grade	HBsAg	Tumor size	CP score	LNM	Risk index	Risk index range		Estimated RFS
						3-year	5-year	
1	Positive	≤ 5 cm	A	Absent	0	0 – 53	0 – 46	50% – 100%
2-1	Positive	> 5 cm	A	Absent	54	54 – 95	47 – 89	30% – 50%
2-2	Negative	≤ 5 cm	A	Absent	55			
2-3	Positive	≤ 5 cm	A	Present	64			
3-1	Positive	≤ 5 cm	B	Absent	100	96 – 117	90 – 111	20% – 30%
3-2	Negative	> 5 cm	A	Absent	109			
4-1	Positive	> 5 cm	A	Present	118	118 – 273	112 – 273	0% – 20%
4-2	Negative	≤ 5 cm	A	Present	119			
4-3	Positive	> 5 cm	B	Absent	154			
4-4	Negative	≤ 5 cm	B	Absent	155			
4-5	Positive	≤ 5 cm	B	Present	164			
4-6	Negative	> 5 cm	A	Present	173			
4-7	Negative	> 5 cm	B	Absent	209			
4-8	Positive	> 5 cm	B	Present	218			
4-9	Negative	≤ 5 cm	B	Present	219			
4-10	Negative	> 5 cm	B	Present	273			

*RFS* relapse-free survival, *ICC* intrahepatic cholangiocarcinoma, *HBsAg* hepatitis B surface antigen, *CP*: Child-Pugh, *LNM* lymph node metastasis

### Validation of the Renji nomogram

In the validation cohort, the median month for the follow-up investigation was 52 months (range, 16 to 81 months) and the 1, 3-year., and overall recurrence rates were 29.2, 68.8, and 74.0%, respectively. The C-index of the risk stratification system for predicting RFS was 0.65 (95% CI, 0.59 to 0.71), which was found to be significantly higher than the AJCC staging system (C-index, 0.57; 95% CI, 0.50 to 0.64), and the calibration curve revealed acceptable agreement between actual and predicted RFS (Fig. 2b).

### Comparison between the Renji nomogram and the AJCC Staging system

**Fig. 3:**
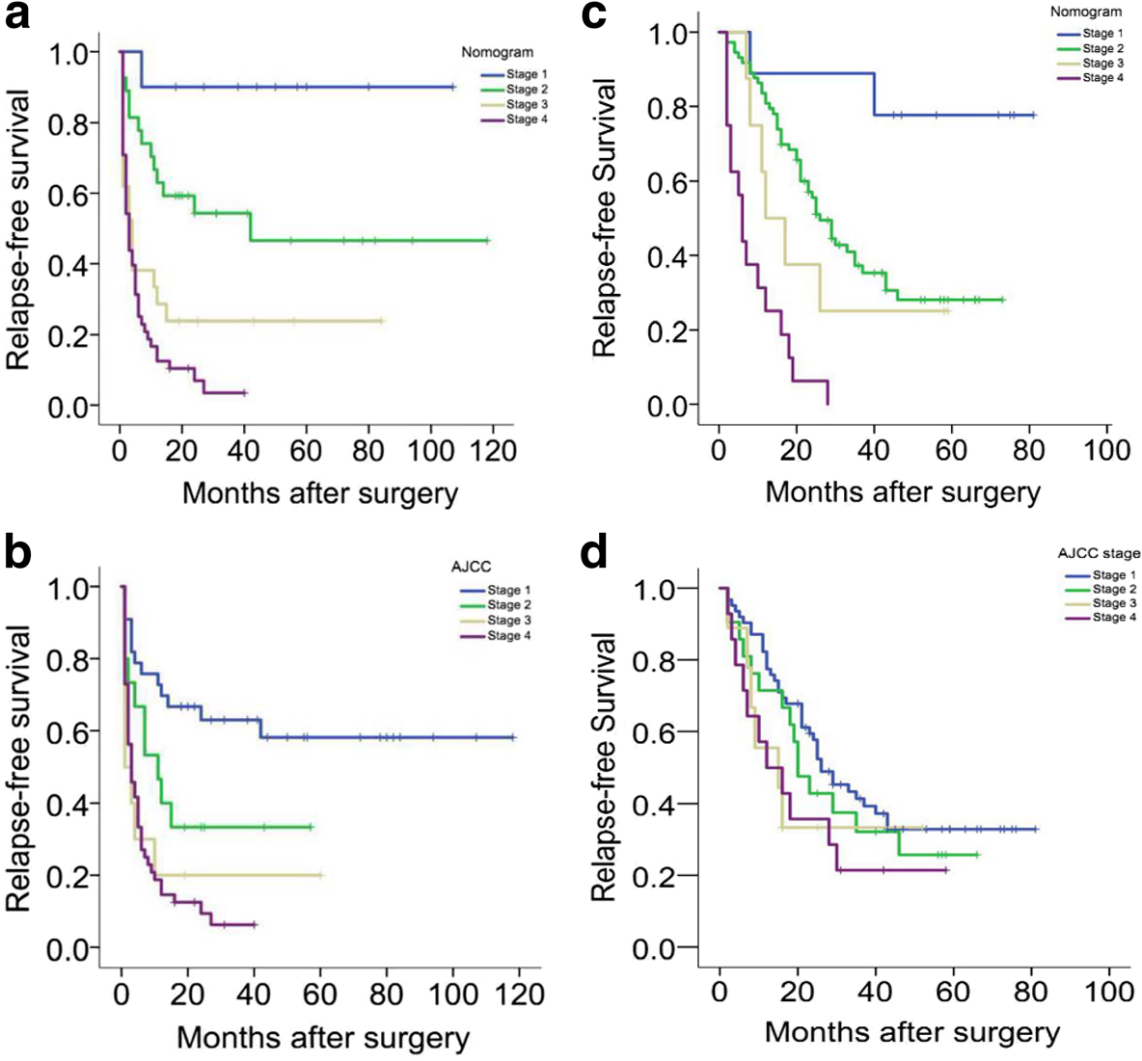
Kaplan-Meier curves of the primary cohort ([**a**] Renji nomogram and [**b**] AJCC staging system) and the validation cohort ([**c**] Renji nomogram and [**d**] AJCC staging system)

Both the Renji nomogram (Fig. 3a) and the AJCC staging system (Fig. 3b) demonstrated excellent prognostic stratification (P [log-rank test] < 0.0001; P [trend] < 0.0001). The nomogram indicated higher precision in predicting 1, 3, and 5-year. RFS in the patients with ICC (C-index, 0.71 vs. 0.66; Table 4). However, actual 3. and 5-year. RFS of the patients in the stage 3 demonstrated relatively low survival outcomes (3, and 5-year. RFS of the Renji nomogram, 15.8 and 5.9%; 3. and 5-year. RFS of the AJCC staging system, 11.1 and 11.1%). In addition, the AJCC staging system could not accurately stratify the patients according to different risk of recurrence in the validation set (Fig. 3c and d).

**Table 4:** Relapse-free survival of the patients according to Renji nomogram and AJCC staging system

Staging system	n (%)	Relapse-free survival (%)					
		CB	Events	1-year	3-year	5-year	C-index
Renji nomogram							0.71
Grade 1	10 (9.4)	9	1	90.0	87.5	75.0	
Grade 2	27 (25.5)	14	13	66.7	40.0	27.8	
Grade 3	21 (19.8)	5	16	33.3	15.8	5.9	
Grade 4	48 (45.3)	3	45	16.7	2.2	0	
AJCC							0.66
Grade 1	33 (31.1)	20	13	72.7	55.6	40.9	
Grade 2	15 (14.2)	5	10	46.7	16.7	0	
Grade 3	10 (9.4)	2	8	20.0	11.1	11.1	
Grade 4	48 (45.3)	4	44	18.8	2.2	0	

*AJCC* American Joint Committee on Cancer, *CB* censored subjects

## Discussion

Most patients with ICC present with advanced disease at the time of diagnosis due to lack of typical clinical manifestations in early stages. Despite surgical approaches, recurrence is more frequent than other malignancies, such as hepatocellular carcinoma, resulting in much more less than a half of patients achieving favorable long-term prognosis [10]. Current perspectives on stratifying patients into stages mainly focus on TNM factors which were proven to significantly influence prognosis of patients with ICC [11]. However, some recent studies indicated that additional risk factors, such as HBV infection, were also found to be significantly associated with prognosis of patients with ICC after hepatic resection [12, 13]. In the present study, we performed univariate analyses on all potential factors that might have affected recurrence of the tumor and included all the variables that showed significant association with the recurrence of the tumor in developing the nomogram, and demonstrated excellent accuracy in predicting recurrence of ICC after hepatic resection (C-index, 0.71; 95% CI, 0.65 to 0.77). Furthermore, unlike previous nomograms that predicted overall survival outcomes, the purpose of our nomogram was to precisely stratify patients according to risk index for recurrence of ICC and exclude irrelevant factors, such as surgical complications, which would lead to unfavorable survival outcomes [6, 14].

The earliest nomogram for ICC was developed in 2013 by Wang et al. [6] on 367 patients with ICC who received partial hepatectomy from 2002 to 2007. Their nomogram was based on the independent factors for overall survival, including serum level of CA19-9, tumor size, number of tumor, vascular and direct invasion, and lymph node and local extrahepatic metastasis. The C-index for the prediction of survival was 0.74, which was found to be higher than AJCC, Nathan, Liver Cancer Study Group of Japan, and Okabayashi staging systems, thereby proving prognostic accuracy of nomogram. The second nomogram was built on basis of 514 patients from international 13 major hepatobiliary centers [14]. The nomogram included age at the time of diagnosis, number and size of tumor, presence of cirrhosis, nodal status, and vascular invasion, which is an initial nomogram that included additional factors rather than TNM factors. However, they put the presence of underlying cirrhosis into the nomogram as an important factor, regardless of its no significant association with survival outcomes in the univariate analysis (*P* = 0.19) and the highest HR of 2.10 (95% CI, 1.46 to 3.01) was found in macrovascular invasion. More recently, Doussot et al. [15] validated the above two nomograms in 188 patients with a median follow-up period of 41 months and reported that both nomograms provided accurate estimation of prognosis (C-index, 0.72 and 0.66), indicating that current nomograms are sufficient to predict overall survival of patients with ICC. In the present study, our data revealed that seropositivity of HBsAg is associated with decreased incidence of recurrence. In addition, tumor size larger than 5 cm, Child-Pugh score of B, and the presence of lymph node metastasis at the time of surgery were associated with more frequent relapse of the tumor. Our nomogram comprehensively included all the above mentioned independent prognostic factors related to the recurrence of ICC, which have not been included in any other staging systems or nomograms.

There has been a controversy on survival outcomes between patients with HBV-associated ICC and patients without HBV infection [16–20]. Several studies demonstrated that HBV infection is a favorable prognostic factor in patients with ICC who underwent hepatic resection [17]. Unfortunately, the presence of HBV infection was excluded in Wang’s nomogram. On the contrary, Tao et al. [21] indicated that HBV infection negatively influenced prognosis of patients with ICC. Another publication from Korea showed that there was no impact of HBV infection on survival outcomes of patients with ICC, and they suggested that early diagnosis of unexpected ICC in HBV carriers might result in relatively favorable outcomes [22]. However, most recently, Wang et al. [23] found that HBV-associated ICC patients achieved significantly better survival outcomes than the patients with hepatolithiasis-associated ICC by analyzing 731 patients with ICC undergoing R0 hepatic resection, and suggested that HBV infection might influence biological malignant invasiveness of the tumor rather than stages of ICC. Comprehensively, a recent meta-analysis demonstrated that HBV infection is a favorable prognostic factor that significantly improves survival outcomes in patients with ICC [24]. The meta-analysis also revealed that HBV infection was associated with underlying cirrhosis (odds ratio [OR], 6.44; 95% CI, 4.33 to 9.56), capsule formation (OR, 6.04; 95% CI, 3.56 to 10.26), and infrequent lymph node metastasis (OR, 0.39; 95% CI, 0.25 to 0.58). Most recently, Hiroya et al. [25] indicated that the absence of viral hepatitis was associated with more frequent incidence of lymph node metastasis in ICC, which led to poor prognosis. However, their study did not separate HBV and HCV, due to limited number of patients (*n* = 32). As shown in our previous study, we preliminarily hypothesized that decreased lymph node metastasis of HBV-associated ICC might be derived from inhibited mesenchymal-to-lymphatic endothelial transition of cancer-associated fibroblasts in stromal areas, followed by reduced number of microlymphatic vessels participating in cancer-associated lymph node metastasis [26]. However, current biological evidences are insufficient to confirm the existence of mesenchymal-to-lymphatic endothelial transition in ICC, which awaits our future research.

The nomogram also contained Child-Pugh score to be an important factor in the estimation of recurrence. This scoring system is composed of ascites, bilirubin, albumin, prothrombin time, and encephalopathy that were considered to be crucial in evaluating liver reserving functions of patients with chronic liver diseases as well as HCC [27]. In previous years, the application of Child-Pugh score to ICC was not established in our center due to the following reasons: (1) liver dysfunction of patients with ICC is partially attributed to the tumor lesions, (2) cirrhosis is uncommon in typical ICC, and (3) subjective evaluation of ascites and encephalopathy. In the present study, merely 6 patients were found to be Child-Pugh score of B and no patient manifested encephalopathy, thereafter actually only 4 variables were included. On the other hand, a previous case-control study of 160 patients with ICC and 2498 patients with HCC by Lee et al. [28] indicated that HBV-associated ICC shared common disease process with HCC in terms of carcinogenesis. In addition, our previous publication indicated that HBV-associated ICC is a different entity with distinctive clinicopathological characteristics, including preponderance of male and younger patients, frequent elevation of serum aspartate transaminase and AFP, and a relatively lower level of CA19-9 [26]. Therefore, as our results indicated, the Child-Pugh scoring system needs to be applied to more precisely predict recurrence of ICC.

However, there are a few underlying limitations that remain to be further confirmed. Firstly, limited number of patients might need an additional modification by future large-scaled studies. Secondly, all the involved patients were from Eastern Asia, so the nomogram needs to be further validated by patients from other regions. Thirdly, specific region of lymph node metastasis was not collected due to retrospective collection of data. Despite the above limitations, our nomogram represents the first stratification system to predict recurrence of ICC after hepatic resection on the basis of Eastern population.

## Conclusions

The nomogram could precisely stratify the patients according to the risk index for the recurrence of ICC by applying seropositivity of HBsAg, tumor size, Child-Pugh score, and lymph node metastasis. The accuracy of the nomogram revealed to be higher than the AJCC staging system in both the study set (C-index, 0.71 vs. 0.66) and the validation set (C-index, 0.65 vs. 0.57). According to the retrospective nature and limited number of patients, our risk stratification system calls for modification by large-sized studies.

## Abbreviations

AFP: Alpha fetoprotein
AJCC: American Joint Committee on Cancer
CA: Carbohydrate antigen
CI: Confidence interval
C-index: Concordance index
HBsAg: Hepatitis B surface antigen
HBV: Hepatitis B virus
HCV: Hepatitis C virus
HR: Hazard ratio
ICC: Intrahepatic cholangiocarcinoma
IQR: Interquartile range
RFS: Relapse-free survival
SD: Standard deviation
